# CD8^+^ T-cell Cytotoxic Capacity Associated with Human Immunodeficiency Virus-1 Control Can Be Mediated through Various Epitopes and Human Leukocyte Antigen Types

**DOI:** 10.1016/j.ebiom.2014.12.009

**Published:** 2014-12-22

**Authors:** Stephen A. Migueles, Daniel Mendoza, Matthew G. Zimmerman, Kelly M. Martins, Sushila A. Toulmin, Elizabeth P. Kelly, Bennett A. Peterson, Sarah A. Johnson, Eric Galson, Kate O. Poropatich, Andy Patamawenu, Hiromi Imamichi, Alexander Ober, Catherine A. Rehm, Sara Jones, Claire W. Hallahan, Dean A. Follmann, Mark Connors

**Affiliations:** a Laboratory of Immunoregulation, National Institute of Allergy and Infectious Diseases, National Institutes of Health, Bethesda, MD, USA; b Clinical Monitoring Research Program, Leidos Biomedical Research, Inc., Frederick, MD, USA; c Biostatistics Research Branch, National Institute of Allergy and Infectious Diseases, National Institutes of Health, Bethesda, MD, USA

**Keywords:** Long-term nonprogressors/elite controllers, Immune control, CD8^+^ T cells, Cytotoxic capacity, Epitope specificity

## Abstract

Understanding natural immunologic control over Human Immunodeficiency Virus (HIV)-1 replication, as occurs in rare long-term nonprogressors/elite controllers (LTNP/EC), should inform the design of efficacious HIV vaccines and immunotherapies. Durable control in LTNP/EC is likely mediated by highly functional virus-specific CD8^+^ T-cells. Protective Human Leukocyte Antigen (HLA) class I alleles, like B*27 and B*57, are present in most, but not all LTNP/EC, providing an opportunity to investigate features shared by their HIV-specific immune responses. To better understand the contribution of epitope targeting and conservation to immune control, we compared the CD8^+^ T-cell specificity and function of B*27/57^neg^ LTNP/EC (n = 23), B*27/57^pos^ LTNP/EC (n = 23) and B*27/57^neg^ progressors (n = 13). Fine mapping revealed 11 previously unreported immunodominant responses. Although B*27/57^neg^ LTNP/EC did not target more highly conserved epitopes, their CD8^+^ T-cell cytotoxic capacity was significantly higher than progressors. Similar to B*27/57^pos^ LTNP/EC, this superior cytotoxicity was mediated by preferential expansion of immunodominant responses and lysis through the predicted HLA. These findings suggest that increased CD8^+^ T-cell cytotoxic capacity is a common mechanism of control in most LTNP/EC regardless of HLA type. They also suggest that potent cytotoxicity can be mediated through various epitopes and HLA molecules and could, in theory, be induced in most people.

## Highlights

• HIV-specific cytotoxicity associated with control can be mediated across a wide variety of epitopes and HLA types.
• Targeting of conserved epitopes does not differentiate patients with immunologic control of HIV-1.
• High level cytotoxic capacity is a feature shared among LTNP/EC across HLA types.

## 1 Introduction

Investigations performed in long-term nonprogressors/elite controllers (LTNP/EC) have provided considerable insights into the mechanisms underlying durable control over HIV replication. Strong associations have been identified between this phenotype and particular Major Histocompatibility Complex (MHC) class I alleles, especially B*27 and B*57 (Kaslow et al., 1996, Migueles and Connors, 2010, Migueles et al., 2000). A similar phenotype has been found in Simian Immunodeficiency Virus (SIV)-infected Rhesus macaques and is associated with Mamu B*8 and B*17 (Loffredo et al., 2007, Yant et al., 2006).

It has been suggested that these protective alleles mediate their effect by presenting peptides whose sequences are conserved due to structural or functional constraints on the virus (Allen et al., 2005, Brockman et al., 2007, Crawford et al., 2007, Friedrich et al., 2004, Goulder et al., 1997, Leslie et al., 2004, Pereyra et al., 2014, Peyerl et al., 2004). In some studies of progressors, focused targeting by HIV-specific CD8^+^ T-cell responses of more conserved regions has been associated with lower HIV RNA levels (Dinges et al., 2010, Kunwar et al., 2013, Liu et al., 2009, Mothe et al., 2011). Although the role of epitope conservation in the effect of MHC on HIV control among progressors is not yet clear, it appears less likely that it differentiates progressors from LTNP/EC bearing protective alleles. In larger groups of B*57^pos^ patients that include true LTNP/EC, the prevalence of epitope sequence variations was comparable between LTNP/EC and progressors (Bailey et al., 2006, Migueles et al., 2003, Miura et al., 2009). In both groups, the CD8^+^ T-cell response targets epitopes restricted by these protective class I proteins (Altfeld et al., 2003, Goulder et al., 1996, Migueles et al., 2000). Nonetheless, most HIV-infected individuals bearing protective alleles experience progressive infection. This suggests that protective genotypes and preferential epitope targeting are clearly not sufficient for high-level HIV control and do not distinguish LTNP/EC from progressors bearing protective alleles (Bailey et al., 2006, Migueles et al., 2000, Migueles et al., 2003, Miura et al., 2009).

In contrast, there is a growing consensus that durable control among patients bearing protective alleles is associated with superior CD8^+^ T-cell function (reviewed in Hersperger et al., 2011). Among the CD8^+^ T-cell functions that have most consistently distinguished LTNP/EC from progressors are increased polyfunctionality, proliferation, loading of cytotoxic proteins, virus suppressive ability and cytotoxic capacity (Betts et al., 2006, Ferre et al., 2009, Hersperger et al., 2010, Migueles et al., 2002, Migueles et al., 2008, Saez-Cirion et al., 2007, Zimmerli et al., 2005). Similarly, there is some evidence of better CD8^+^ T-cell functionality in LTNP/EC macaques compared to progressors (Mendoza et al., 2013).

A better understanding of the contributions of epitope targeting and conservation could potentially be obtained by investigating features of the response shared between LTNP/EC with and without protective alleles. Depending upon the case definition used, 59–79% of LTNP/EC bear HLA B*27 or B*57 (Migueles and Connors, 2010). Thus far, the CD8^+^ T-cell response of the remaining individuals has been anecdotally reported and not well characterized (Hersperger et al., 2010, Lecuroux et al., 2014, Migueles et al., 2002, Migueles et al., 2008, Migueles et al., 2009, Saez-Cirion et al., 2007, Saez-Cirion et al., 2009). In the present study, we analyzed the epitope specificity in a cohort of B*27/57^neg^ LTNP/EC to provide greater insight into the mechanisms of control over HIV replication. The responses in B*27/57^neg^ LTNP/EC recognized epitopes restricted by a variety of HLA class I proteins similar to those of B*27/57^neg^ progressors. These epitopes were not distinguished by their conservation, but rather, varied to the same degree as those restricted by other alleles. CD8^+^ T-cell mediated killing of autologous HIV-infected targets was the parameter shared between LTNP/EC with and without the B*27 or B*57 protective alleles. This cytotoxicity was mediated through HLA proteins that are highly prevalent, raising the possibility that vaccines or immunotherapies that might induce cytotoxic function could do so in a large portion of the population.

## 2 Experimental Procedures

### 2.1 Subjects

The NIAID Institutional Review Board approved this study. All subjects were adults who provided written informed consent following detailed protocol review with the Principal Investigator. PBMC were collected by leukapheresis. HIV infection was determined by HIV-1/2 immunoassay (Abbott Laboratories, Abbott Park, IL) and Cambridge Biotech HIV-1 Western blot (Maxim Biomedical, Inc., Rockville, MD). All subjects acquired HIV infection in the USA and, therefore, are likely infected with clade B viruses. LTNP/EC, including those not carrying the classically protective HLA class I alleles B*27 and B*57 (n = 23) and a randomly selected group of B*27/57^pos^ LTNP/EC (n = 23), were clinically healthy and had stable CD4^+^ T-cell counts, HIV-1 RNA levels < 50 copies/mL, no ART and no history of opportunistic diseases. Slow (n = 6) and chronic (n = 7) progressors had detectable HIV-1 RNA levels (> 1000–< 10,000 or > 10,000 copies/mL, respectively) and declining CD4^+^ T-cell counts without ART, as previously defined (Migueles et al., 2008). HLA class I/II typing was performed by sequence-specific hybridization (Migueles et al., 2000).

### 2.2 Optimal Epitope Determination of Immunodominant HIV-specific CD8^+^ T-cells

Intracellular cytokine detection assays using cryopreserved peripheral blood mononuclear cells (PBMC) stimulated for 6 h with synthetic peptides homologous with consensus clade B, HIV-1 sequences (final concentration, 2 μg/mL; NIH AIDS Research and Reference Reagent Program, Germantown, MD) were performed as previously described (Migueles et al., 2009). Responses above background were further mapped with smaller peptide pools and individual 15-mer peptides. In determining response breadth, recognition of two or more adjacent 15-mer peptides was addressed according to a previously published algorithm (Frahm et al., 2004).

Determination of optimal epitopes was performed with synthesized truncations of the targeted 15-mer by sequential amino acid elimination from either the N- or C-terminus resulting in 8–14 amino-acid long peptides (Peptide 2.0, Inc., Chantilly, VA) (Hansen et al., 2013). HLA restriction was confirmed with peptide-pulsed, heterologous target PBMC matched at a single HLA allele. Immunodominant responses were the highest frequency responses (when rank ordered from highest to lowest) that comprised at least 50% of the total magnitude in a single patient or were detected among multiple individuals with the same HLA type. All others were considered non-immunodominant. A response was considered new if the epitope had not been previously reported or if the restricting HLA differed from prior reports. “Predicted” responses were included if at least two of the following three criteria were met: (1) a known epitope within the recognized protein sequence and a restricting HLA matching one from the submitted haplotype were located within the HIV immunology database by the LANL Epitope Location Finder (ELF; www.hiv.lanl.gov/content/sequence/ELF/epitope_analyzer.html); (2) a potential epitope possessed one or more anchor residue motifs associated with the submitted HLA alleles as determined by Motif Scan (www.hiv.lanl.gov/content/immunology/motif_scan/motif_scan); or (3) a potential epitope within the recognized peptide sequence was predicted by the Immune Epitope Database (IEDB; tools.immuneepitope.org/mhci) or Propred-I (www.imtech.res.in/raghava/propred1) algorithms to bind strongly with one of the submitted HLA proteins at conservative 2% thresholds.

### 2.3 Shannon Entropy Score

Shannon entropy of HIV-1 peptides, used as a measure of variability at the population level (Frahm et al., 2004), was derived by averaging the entropy scores of the 8–15 amino acids comprising a particular peptide, which had been averaged for each amino acid residue from clade B protein alignments in the LANL database. Entropy scores were determined for best-defined (“A list”) CTL/CD8^+^ epitopes reported in LANL (www.hiv.lanl.gov/content/immunology/tables/optimal_ctl_summary), epitopes known to be restricted by B27/57 from LANL and epitopes targeted by the immunodominant responses of the B*27/57^neg^ patients in this study. These were compared with the entropy of the major HIV-1 gene products by randomly selecting 1000 sequences each from Nef, Gag and Pol sequences of lengths 8, 9, 10 and 11 amino acids in proportion to the epitope lengths of the 3 combined epitope groups (i.e., 13%, 8-mers; 60%, 9-mers; 17%, 10-mers; and 10%, 11-mers). Entropy scores were also determined for Gag overlapping 15-mer peptides and correlated with the prevalence of individuals recognizing a given 15-mer.

### 2.4 HIV-specific CD8^+^ T-cell Cytotoxic Capacity

Cytotoxicity of autologous HIV_SF162_-infected CD4^+^ T-cell targets by CD8^+^ T-cells that had been stimulated for 6 days was assessed by GrB target cell activity and ICE, as described previously (Migueles et al., 2008, Migueles et al., 2009). To assess per-cell cytotoxic capacity, responses were plotted against the true E/T ratios for each patient and the generated group curves were compared, as described previously (Migueles et al., 2008, Migueles et al., 2009). Cells were combined at a plated E/T of 25:1. True E/T ratios were derived from parallel measurements of the frequencies of HIV-specific CD8^+^ T cells based on IFN-γ production (true effector cell numbers) and the percentages of HIV p24^+^ T-cell targets (true target cell numbers). To confirm the epitope specificities of the immunodominant cytotoxic responses, GrB activity was measured after co-incubating day 6 effectors for 1 h with autologous, un-infected CD4^+^ T-cell targets pulsed with either the immunodominant epitopes found in the intracellular cytokine assays or an irrelevant peptide. To confirm the contribution of HLA class I restriction to the total cytotoxic responses, GrB activity and ICE were measured after co-incubating day 6 effectors with fresh heterologous un-infected or HIV_SF162_-infected targets matched at a single HLA class I allele. Immunodominant responses were among the highest responses and contributed ≥ 50% to the magnitude of the total cytotoxic response in a single patient. To examine per-cell cytotoxic capacity of immunodominant and non-immunodominant responses, these were subsequently plotted against the true E/T ratios of each response based on net true effector cell numbers (following co-incubation of a given patient's effectors with heterologous HIV_SF162_-infected CD4^+^ T-cell targets matched at a single HLA locus minus background responses to heterologous un-infected targets) and the true numbers of heterologous HIV p24^+^ T-cell targets.

### 2.5 Flow Cytometry

Multiparameter flow cytometry was performed according to standard protocols. All staining was performed at 4 °C for 30 min with the following antibodies (BD Biosciences): FITC-conjugated anti-CD3; PerCP-conjugated anti-CD3 and anti-CD8; Am Cyan-conjugated anti-CD8; APC-conjugated anti-CD4 and anti-IFN-γ; PE-conjugated anti-CD8 and anti-CD69. RD1-conjugated anti-HIVp24 (Kc57; Beckman Coulter, Inc., Brea, CA) was used to confirm target cell infection and measure elimination of p24-expressing cells. Samples were analyzed on a FACSAria multi-laser cytometer (BD Biosciences) with FACSDiva software. Data were analyzed using FlowJo software (TreeStar, San Carlos, CA).

### 2.6 Statistical Analysis

Analysis of covariance was used to quantify the differences in GrB activity and ICE among the various patient groups and among immunodominant versus non-immunodominant responses over the shared range of logged E/T ratios. Comparisons of independent groups were made by the Wilcoxon two-sample test. Correlations were determined by the Spearman rank method. The Bonferroni method was used to adjust p values for multiple testing.

## 3 Results

### 3.1 HLA B*27/57^neg^ LTNP/EC Have Clinical Features Similar to Those of B*27/57^pos^ LTNP/EC

LTNP/EC not bearing the classically protective HLA class I alleles B*27 or B*57 comprised 21% (23/112) of our entire LTNP/EC cohort (Migueles and Connors, 2010) and had similar features to those of 23 randomly selected B*27/57^pos^ LTNP/EC (Table S1). B*27/57^neg^ and B*27/57^pos^ LTNP/EC had similar, profound control over HIV (median HIV RNA levels < 50 copies/mL in both groups; p > 0.5) for similarly long durations of infection (medians, 17 versus 24 years, respectively; p > 0.5) and had comparable CD4^+^ T-cell counts (medians, 913 versus 753 cells/mm^3^, respectively; p > 0.5). In a group of 13 B*27/57^neg^ viremic progressors used as controls, viral RNA levels were significantly higher (median, 10,443 copies/mL, p < 0.001 for all comparisons) and CD4^+^ T-cell counts were significantly lower (median, 461; p ≤ 0.001 for all comparisons; Table S1) compared to LTNP/EC. Despite small sample sizes, the composition of the two LTNP/EC sub-groups also appeared to be similar with respect to gender (p > 0.5), race/ethnicity (p > 0.5) and mode of HIV acquisition (p > 0.5).

Fifteen out of 23 (65%) of the B*27/57^neg^ LTNP/EC carried at least one weakly protective HLA allele previously demonstrated to be enriched in LTNP/EC, including B*13, *15*, *44*, *51*, *58* and *81* (Flores-Villanueva et al., 2001, Frahm et al., 2006, Goulder et al., 2000, Honeyborne et al., 2007, Kaslow et al., 1996, Zhang et al., 2011). However, no particular HLA type predominated among these 23 B*27/57^neg^ LTNP/EC. In addition, 8/23 B*27/57^neg^ LTNP/EC had HLA alleles considered to be neutral or deleterious regarding their impact on disease progression.

### 3.2 Immunodominant CD8^+^ T-cell Responses of B*27/57^neg^ LTNP/EC Are Restricted by Various HLA Proteins

**Fig. 1:**
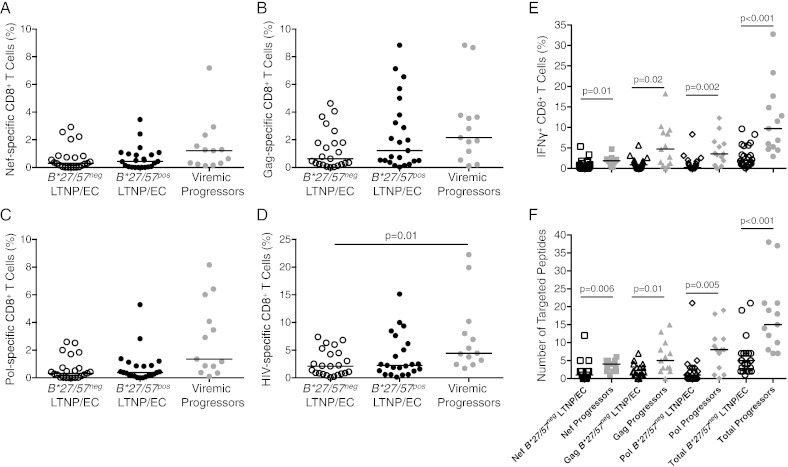
HIV-specific CD8^+^ T-cell responses of B*27/57^neg^ LTNP/EC are similar to those of B*27/57^pos^ LTNP/EC. Frequencies of IFN-γ^+^CD69^+^CD8^+^ T-cells in response to 15-mer peptide pools spanning HIV-1 (A) Nef, (B) Gag, (C) Pol and (D) total (sum of A–C) are shown for B*27/57^neg^ LTNP/EC (n = 23), B*27/57^pos^ LTNP/EC (n = 23) and B*27/57^neg^ viremic progressors (n = 13). Upon further mapping, (E) the sum of the frequencies of IFN-γ^+^CD69^+^CD8^+^ T-cells and (F) the sum of the numbers of targeted peptides are shown for B*27/57^neg^ LTNP/EC and B*27/57^neg^ viremic progressors. Horizontal lines represent median values. Comparisons were made using the Wilcoxon two-sample test. Only significant p values are shown.

To understand the contribution of epitope targeting to immunologic control in B*27/57^neg^ LTNP/EC, individual specificities were mapped and the HLA molecule they bound was determined. Pools of synthetic 15-mer peptides, overlapping by 10 amino acids (see Experimental Procedures) and spanning the major HIV-1 gene products Nef, Gag and Pol were used in intracellular cytokine assays (Fig. 1). The magnitude of the gene product-specific CD8^+^ T-cell responses was variable and tended to be highest for Gag, as observed in prior studies (Betts et al., 2001, Migueles et al., 2000, Migueles et al., 2002, Saez-Cirion et al., 2007). Although the responses tended to be higher for 13 viremic progressors (untreated slow progressors and typical progressors combined), the gene product-specific and total HIV-specific CD8^+^ T-cell responses were not significantly different between 23 B*27/57^neg^ and 23 B*27/57^pos^ LTNP/EC (p > 0.05 for all comparisons; Fig. 1A–D).

Using smaller pools and individual peptides, virus-specific CD8^+^ T-cell responses were further mapped down to the individual 15-mers (Tables S2–4 and Fig. 1E, F). In this analysis, the frequencies of the total responses targeting 15-mer peptides were significantly lower in 22 B*27/57^neg^ LTNP/EC than 13 B*27/57^neg^ progressors (2.0% versus 9.7%, p < 0.001; Fig. 1E), consistent with the responses measured to the peptide pools (Fig. 1A–D). Moreover, fewer individual 15-mer peptides were targeted by the CD8^+^ T-cell responses of B*27/57^neg^ LTNP/EC than B*27/57^neg^ progressors (median 5 versus 15, p < 0.001; Tables S2–4 and Fig. 1F). In summary, the HIV-specific CD8^+^ T-cell responses of B*27/57^neg^ LTNP/EC were readily detectable, variable in magnitude and narrow in breadth (Migueles et al., 2000, Migueles et al., 2002).

To further resolve the specificities of the immunodominant HIV-specific CD8^+^ T-cell responses of B*27/57^neg^ patients, responses targeting individual 15-mer peptides were fine mapped if their frequency was ≥ 0.2% and if they were either among the highest of a given patient or were recognized by multiple patients. Fine mapping was performed with synthesized truncations, 8–14 amino acids long, of the recognized 15-mer peptides in intracellular cytokine assays. Once the optimal epitopes were determined, HLA class I restriction was confirmed in the same assays with peptide-pulsed, heterologous targets matched at a single HLA locus. When possible, optimal epitopes and/or HLA restriction elements were predicted for unmapped dominant responses (see Experimental Procedures). Epitope-specific CD8^+^ T-cells ranged in frequency from 0.2 to 4.3% (Table 1, Table 2). Remarkably, many B*27/57^neg^ patients targeted specificities not previously described. Eleven confirmed responses were new: 5 in Nef (B8-SA9, B14-AL9, B58-AL9, B58-GY9 and C2-GY9), 2 in Gag (B40-DG11 and B40-LI10) and 4 in Pol (B38-YW9, B73-IP9, C5-HT9 and C8-VL8; Table 1, Table 2). These were immunodominant in 14 B*27/57^neg^ patients: 8 LTNP/EC, 4 slow progressors and 2 chronic progressors (Table 2). Among B*27/57^neg^ LTNP/EC, some responses were restricted by class I proteins that have been enriched, albeit less so than B27 and B57, in LTNP/EC cohorts, such as, B15, B44, B51, B58 and B81 (Flores-Villanueva et al., 2001, Frahm et al., 2006, Goulder et al., 2000, Kaslow et al., 1996, Zhang et al., 2011). However, these same responses were also identified in progressors. B51-restricted responses were particularly immunodominant with frequent recognition of the LI9, RI8 and NI9 epitopes (Table 2) (Zhang et al., 2011). Interestingly, many responses in LTNP/EC were restricted by historically neutral, deleterious or uncommon HLA proteins, such as B8, B35 and B73, respectively. The rare B*73 allele was present in only 4 of the 2020 HLA typed, HIV-positive patients screened to date in our outpatient clinic, yet B73 restricted the most immunodominant response of LTNP/EC-8. In addition, HLA-C proteins (e.g., C1, C3, C5 and C8) restricted some dominant responses. The immunodominance of HLA-C1-restricted responses is consistent with prior observations (Buranapraditkun et al., 2011) and might be due to higher surface expression and better antigen presentation by particular HLA-C proteins (Thomas et al., 2009).

Remarkably, some immunodominant epitopes were cross-restricted by different HLA proteins in different patients. For example, the highly conserved TL9 epitope (Goulder et al., 2000, Liu et al., 2013) was restricted by B42 in two LTNP/EC, B81 in two other LTNP/EC, and C8 in a slow progressor (Table 2). In only 7/67 total confirmed or predicted immunodominant responses were epitopes, known to also be restricted by B27 or B57, targeted: 5 confirmed responses in Nef (B14-AL9 of LTNP/EC-11, B58-AL9 of LTNP/EC-7, C3-AL9 of LTNP/EC-10, B15-LL9 of LTNP/EC-13 and B58-HW9 of SP-1); 1 predicted response in Gag (B58-IW9 of SP-1); and 1 predicted response in Pol (B58-SW10 of LTNP/EC-6; Table 1, Table 2) (Frahm et al., 2005, Goulder et al., 1996). Of immunodominant HIV-specific CD8^+^ T-cell responses of the B*27/57^neg^ patients in this study, more than 2/3 were not directed against the same or overlapping sequences as those restricted by B27 or B57. Rather, these were scattered over the HIV proteome and distal to known B27- or B57-restricted responses. These data suggest that targeting areas within or close to those typically targeted by B*27/57^pos^ LTNP/EC is not a requirement for immunologic control of HIV-1.

### 3.3 Immunodominant Responses of B*27/57^neg^ LTNP/EC Target Peptides Not Differentiated by Entropy

**Fig. 2:**
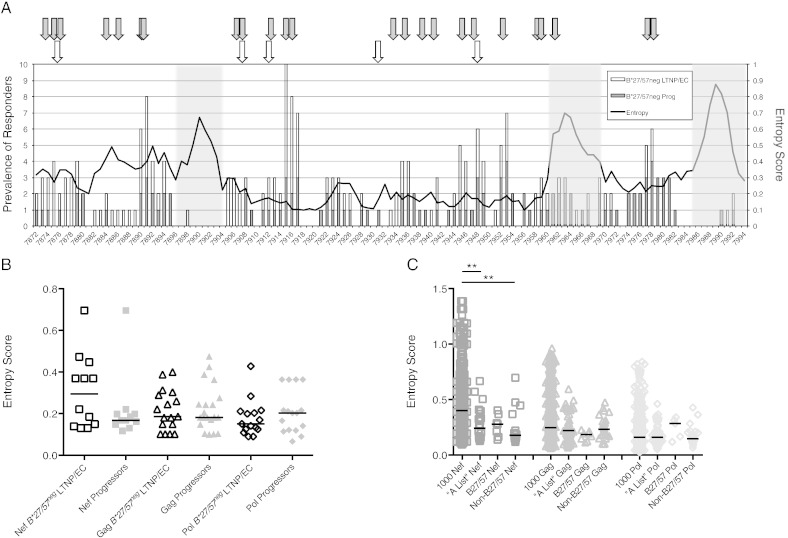
Immunodominant responses of both B*27/57^neg^ LTNP/EC and progressors target conserved regions in HIV-1. (A) Prevalence of B*27/57^neg^ LTNP/EC (n = 23, white bars) and B*27/57^neg^ progressors (n = 13, solid gray bars) with significant IFN-γ^+^CD69^+^CD8^+^ T-cell responses above background to individual Gag 15-mers are shown. Trend line connects the Shannon entropy scores for each 15-mer. Block arrows identify sequence positions corresponding to B27/57-restricted Gag epitopes (B27-KK10, B57-IW9, B57-KF11, B57-TW10 and B57-QW9; white) and mapped Gag epitopes targeted by immunodominant CD8^+^ T-cell responses of B*27/57^neg^ subjects (n = 36, gray). Highly variable regions are shaded. (B) Entropy scores of confirmed and predicted Nef (squares), Gag (triangles) and Pol (diamonds) epitopes targeted by immunodominant CD8^+^ T-cell responses of B*27/57^neg^ LTNP/EC (open symbols) and progressors (solid gray symbols) are shown. (C) Entropy scores of 1000 pseudo-epitopes in Nef, Gag and Pol compared with those of best-defined (“A list”) epitopes and known B27/57-restricted epitopes from LANL and epitopes recognized by B*27/57^neg^ subjects (non-B27/57) in this study. Horizontal lines represent median values. Comparisons were made using the Wilcoxon two-sample test. Only significant p values are shown as **(p < 0.01).

It has been suggested that the association between B*57 or B*27 alleles and lower viral loads is mediated by targeting functionally critical and, therefore, more highly conserved parts of the proteome (Allen et al., 2005, Brockman et al., 2007, Crawford et al., 2007, Friedrich et al., 2004, Goulder et al., 1997, Leslie et al., 2004, Liu et al., 2013, Peyerl et al., 2004). To examine whether recognition prevalence was related to the degree of peptide conservation, Shannon entropy was used as a measure of HIV-1 peptide variability or diversity (see Experimental Procedures). Lower entropy scores correspond to more conserved peptides (Frahm et al., 2004). Entropy of Gag 15-mers was then plotted with the number of B*27/57^neg^ patients mounting any response above background to each 15-mer peptide (Fig. 2A). Variable recognition of Gag 15-mers was found in both B*27/57^neg^ LTNP/EC and progressors. Although entropy did not correlate with the total number of patients recognizing each 15-mer (p > 0.05), highly variable sequences were targeted infrequently (Fig. 2A). These results suggest that the Gag-specific CD8^+^ T-cells of both B*27/57^neg^ LTNP/EC and progressors were similarly scattered throughout Gag, but tended to spare regions of high entropy (Frahm et al., 2004, Sunshine et al., 2014).

Since 15-mer peptides contain amino acids lying outside of putative epitopes, calculated entropies of the former may not accurately reflect the variability of actual targeted 9-11-mer epitopes. To circumvent this issue, entropy scores were determined for the confirmed and predicted epitopes targeted by the immunodominant CD8^+^ T-cell responses of the B*27/57^neg^ patients in this study (Table 1, Table 2). Considering all Nef, Gag and Pol epitopes, median entropies did not differ significantly between LTNP/EC and progressors (p > 0.05 for all comparisons; Fig. 2B).

To determine whether the variability of epitopes restricted by non-B27/57 proteins differed from that of B27- or B57-restricted epitopes, the entropies of epitopes targeted by the immunodominant responses in this study were compared with entropies of the following peptides: best-defined (“A list”) CTL/CD8^+^ epitopes in the LANL database, those restricted by B27 and B57 in LANL, and pools of 1000 pseudo-epitopes derived from each major gene product (see Experimental Procedures). By this method, the median entropy of total Gag (1000 Gag, 0.25) or Pol (1000 Pol, 0.16) was not significantly different from the epitopes targeted in these gene products (p > 0.05 and p ≥ 0.5 for all comparisons, respectively; Fig. 2C). The median entropy of the more highly variable Nef (1000 Nef, 0.40) was significantly higher than the median entropies of the best-defined Nef epitopes from LANL (0.24; p = 0.004) and Nef epitopes identified in the current study (0.18; p = 0.005) and tended to be higher than known B27/57-restricted Nef epitopes (0.28; p = 0.09). However, the entropies of the Nef epitope groups were comparable to each other (p > 0.5 for all comparisons; Fig. 2C). Thus, the variability of epitopes targeted by the immunodominant virus-specific CD8^+^ T-cell responses of B*27/57^neg^ patients were similar between LTNP/EC and progressors, and no more conserved than epitopes restricted by HLAs previously associated or not associated with immunologic control of HIV-1. These findings suggest that the conservation of targeted epitopes is not a parameter that uniquely identifies patients with immunologic control of HIV-1.

### 3.4 Gag Epitopes of Autologous Viruses Contained Few Variations in B*27/57^neg^ LTNP/EC and Progressors

Immunodominant HIV-specific CD8^+^ T-cell responses of both B*27/57^neg^ LTNP/EC and progressors targeted relatively conserved regions of HIV-1 based on entropy measurements. Even though mutations conferring immune escape or impaired replication capacity have not consistently distinguished B*27/57^pos^ LTNP/EC from progressors (Bailey et al., 2006, Blankson et al., 2007, Buckheit et al., 2012, Lamine et al., 2007, Migueles et al., 2003, Miura et al., 2009, Navis et al., 2007), it remained possible that differences in sequence variations between B*27/57^neg^ LTNP/EC and progressors might account for differential control. Therefore, the autologous *gag* gene was sequenced in 10 B*27/57^neg^ subjects (5 LTNP/EC, 5 progressors). Most regions recognized by the immunodominant responses of these individuals contained few variations and were homologous with the HIV-1 clade B consensus sequence (Table 3). A notable exception was the K9R variant of the LTNP/EC-4's A3-RK9 epitope that has inconsistently been associated with loss of recognition (Bansal et al., 2005, Horton et al., 2006). Infrequent variations involving TCR contact residues were observed, but these do not necessarily confer escape (Bailey et al., 2006, Migueles et al., 2003, Pohlmeyer et al., 2013). No putative upstream or downstream compensatory mutations were found (data not shown). Altogether, these findings suggest sequence variations that might confer escape or reduced replicative capacity do occur in some individuals, but sequence variations do not clearly differentiate most HLA-matched LTNP/EC from progressors (Bailey et al., 2006, Blankson et al., 2007, Buckheit et al., 2012, Lamine et al., 2007, Migueles et al., 2003, Miura et al., 2009, Navis et al., 2007).

### 3.5 HIV-specific CD8^+^ T-cells of B*27/57^neg^ LTNP/EC Mediate Potent Cytotoxicity

**Fig. 3:**
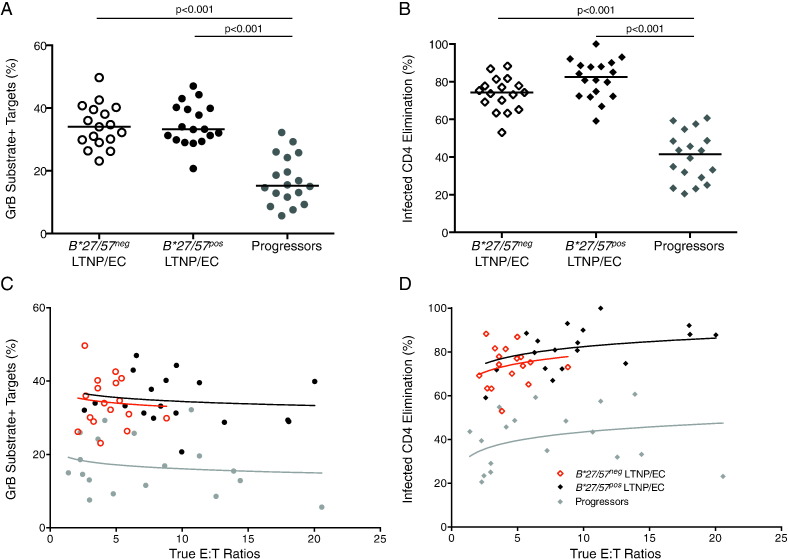
Bulk and per-cell HIV-specific CD8^+^ T-cell cytotoxic responses of B*27/57^neg^ LTNP/EC exceeded those of progressors and were comparable to those of B*27/57^pos^ LTNP/EC. After 1 h co-incubation, CD8^+^ T-cell cytotoxic responses measured by (A) GrB target cell activity (circles) and (B) infected CD4^+^ T-cell elimination (ICE, diamonds) are shown for B*27/57^neg^ LTNP/EC (open symbols, n = 17), B*27/57^pos^ LTNP/EC (solid black symbols, n = 18) and viremic progressors (solid gray symbols, n = 18). Horizontal lines represent median values. Data are representative of three independent experiments. Comparisons were made using the Wilcoxon two-sample test. Only significant p values are shown. Per-cell cytotoxic capacity was assessed in B*27/57^neg^ LTNP/EC (red symbols), B*27/57^pos^ LTNP/EC (black open symbols) and viremic progressors (solid gray symbols) through comparisons of cytotoxicity curves, which were generated by plotting (C) GrB activity and ICE (D) values against the true E/T ratios based upon measurements of IFN-γ^+^ CD8^+^ T-cells and p24-expressing targets. Analysis of covariance was used to quantify the differences in GrB activity and ICE among the groups over the shared range of logged E/T ratios.

Qualitative features of virus-specific CD8^+^ T-cells, such as proliferative and cytotoxic capacities, measured after several days of re-stimulation are far and away the most robust parameters differentiating the responses of B*27/57^pos^ LTNP/EC from progressors (reviewed in Hersperger et al., 2011). HIV-specific CD8^+^ T-cell cytotoxic responses of 17 B*27/57^neg^ LTNP/EC, measured by the median percent of GrB^+^ targets (34%) or infected CD4^+^ T-cell elimination (ICE; 74.3%), were high overall and comparable to those of 18 B*27/57^pos^ LTNP/EC (33.2%, p > 0.5 and 82.6%, p > 0.05, respectively). These cytotoxic responses were significantly higher than those of 18 progressors, including 5 who were B*27/57^pos^ (15.3%, p < 0.001 and 41.4%, p < 0.001, respectively; Fig. 3A, B).

Given the greater proliferative capacity of HIV-specific CD8^+^ T-cells of LTNP/EC compared to progressors, it is critical to additionally measure cytotoxic capacity on a per-cell basis. When the cytotoxic responses of each patient group were plotted against the true measured effector-to-target (E/T) ratios and compared, the cytotoxicity curves of B*27/57^neg^ and B*27/57^pos^ LTNP/EC were superimposable with each other (p > 0.5) and differed significantly over the shared range of E/T from that of progressors (p < 0.001 for all comparisons; Fig. 3C, D). These findings support that the re-stimulated HIV-specific CD8^+^ T-cells of B*27/57^neg^ LTNP/EC exhibit both increased bulk and per-cell cytotoxicity compared to progressors.

**Fig. 4:**
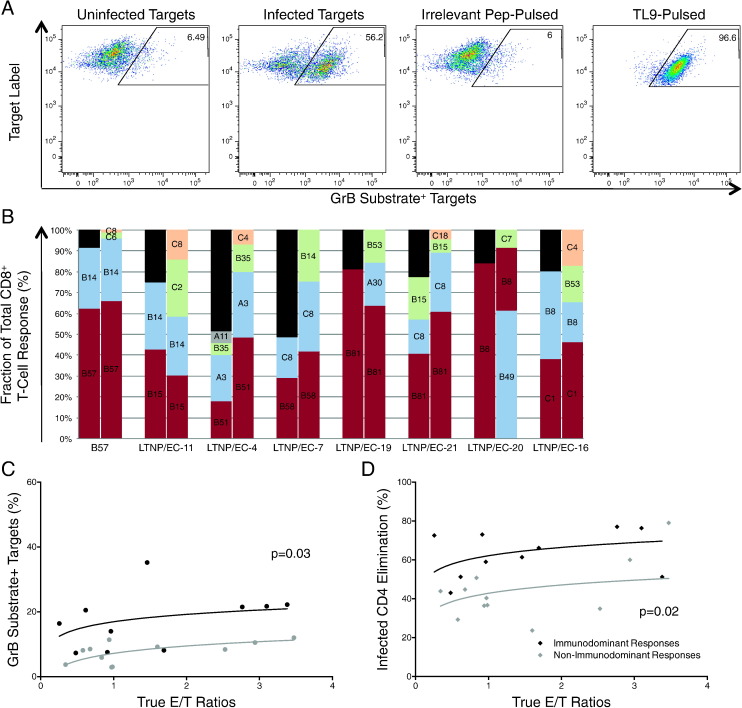
Immunodominant HIV-specific CD8^+^ T-cell responses preferentially expanded and contributed most significantly to overall cytotoxicity. (A) Representative plots depicting GrB activity after 1-hour co-incubation of LTNP/EC-19 CD8^+^ T-cells (initially stimulated for 6 days with autologous HIV_SF162_-infected CD4^+^ T-cells) with un-infected CD4^+^ T-cell targets (background), HIV_SF162_-infected CD4^+^ T-cell targets, un-infected CD4^+^ T-cell targets pulsed with an “irrelevant” peptide (elicited no response in cytokine detection assays) and un-infected CD4^+^ T-cell targets pulsed with the TL9 peptide (LTNP/EC-19's immunodominant epitope in cytokine assays). Numbers in top, right corner correspond to the percentages of the total targets that are GrB substrate^+^ after 1-hour co-incubation with effectors. (B) Cytotoxic responses were further mapped after co-incubation of CD8^+^ T-cells with heterologous HIV_SF162_-infected CD4^+^ T-cell targets matched at a single HLA locus (overlaid on bars) in 7 B*27/57^neg^ and one representative B*27/57^pos^ LTNP/EC (right bars). Background in response to heterologous un-infected targets was subtracted. For comparison, the fractions of the total IFN-γ response restricted by a particular HLA protein are also shown (left bars). Black bars represent unmapped responses. Data are representative of three independent experiments. (C–D) Cytotoxic responses, measured by (C) GrB activity (circles) or (D) ICE (diamonds) from the 7 B*27/57^neg^ LTNP/EC in (B), were categorized as immunodominant (black symbols, n = 10) or non-immunodominant (gray symbols, n = 11), plotted against the true E/T ratios and compared (see Experimental Procedures).

We next sought to determine the peptide specificities and HLA restricting elements responsible for CD8^+^ T-cell-mediated killing of autologous HIV-infected CD4^+^ T-cell targets in B*27/57^neg^ LTNP/EC. First, CD8^+^ T-cell effectors were co-incubated for 1 h with autologous uninfected CD4^+^ T-cell targets pulsed with either irrelevant peptides or epitopes that had induced the highest responses in the intracellular cytokine assays for a particular patient (Table 1). In most instances, greatest cytotoxicity was observed against targets pulsed with peptides recognized by immunodominant responses in intracellular cytokine assays (representative LTNP/EC 19, Fig. 4A). To determine the relative contribution of each HLA restriction element to the total cytotoxic response, expanded HIV-specific CD8^+^ T-cells were co-incubated with heterologous uninfected and HIV-infected CD4^+^ T-cell targets matched at a single locus (Fig. 4B). In 6 of 7 cases (excluding one representative B*57^pos^ LTNP/EC), the HLA class I-restricted CD8^+^ T-cell responses that were immunodominant in the intracellular cytokine assays contributed most significantly to the total cytotoxic response for each individual (Table 1, Table 2 and Fig. 4B). Of note, three alleles previously associated with lower viral loads, B*51, B*58 and B*81, were reproducibly immunodominant. These results suggested that the HLA class I-restricted CD8^+^ T-cell responses determined to be immunodominant in the intracellular cytokine assays were typically the same ones to preferentially expand and mediate most cytotoxicity (Horton et al., 2006, Migueles et al., 2000, Migueles et al., 2002, Migueles et al., 2008).

It remained possible that greater killing through a given HLA molecule for a given patient might simply be due to a higher response frequency. To further understand the relative contribution of cytotoxic capacity and frequency, the 10 immunodominant and 11 non-immunodominant cytotoxic responses from the 7 aforementioned B*27/57^neg^ LTNP/EC were plotted against the measured E/T ratios and compared. The per-cell cytotoxic capacity of immunodominant responses was significantly greater than that of non-immunodominant responses measured by either GrB activity (p = 0.03; Fig. 4C) or ICE (p = 0.02; Fig. 4D). These results suggest that B*27/57^neg^ LTNP/EC share with B*27/57^pos^ LTNP/EC the ability to mediate highly potent cytotoxicity against autologous HIV-infected CD4^+^ T-cells. Further, such responses can be mediated through HLAs not previously associated with protection that are highly prevalent in the human population.

## 4 Discussion

The results of the present study provide several important insights regarding the roles of CD8^+^ T-cell function and specificity in immunologic control of HIV-1 infection. Over the past several years it has been suggested that protective alleles, such as B*27 and B*57, mediate their effect through specificity (Allen et al., 2005, Brockman et al., 2007, Crawford et al., 2007, Friedrich et al., 2004, Goulder et al., 1997, Leslie et al., 2004, Pereyra et al., 2014, Peyerl et al., 2004), which might occur by targeting structurally or functionally critical areas of the proteome. Escape mutations, if they occur, would result in a large fitness cost, constraining viral replication. However, the results of the present study are not consistent with this hypothesis. Many B*27/57^neg^ patients studied here had no alleles even weakly associated with reduced viral load, indicating that a protective HLA background is not required for high-level immune control. Many B*27/57^neg^ LTNP/EC targeted areas of the proteome non-overlapping with, and in most cases quite distal to, areas containing B27/57-restricted peptides. Furthermore, the epitopes they targeted were not distinguished from progressors by greater conservation scores or by a lack or excess of escape mutations. Response features shared between B*27/57^neg^ and B*27/57^pos^ LTNP/EC were killing of autologous CD4^+^ T-cells by CD8^+^ T-cells with potent per-cell cytotoxic capacity. These results suggest that the effect of protective HLAs is not necessarily the result of presentation of particularly constrained epitopes. Rather, effective cytotoxicity associated with immunologic control may be mediated through a broad array of epitopes and HLA molecules.

The results of the present study, to some extent, may narrow the likely explanations for how protective HLAs mediate their effects. They suggest that although favorable HLAs may tilt the balance toward immunologic control of HIV, potent cytotoxicity can be mediated through relatively common epitopes presented by HLAs not associated with protection. HLA-peptide-T-cell receptor interactions might impact the control of viral replication based upon peptide/HLA stoichiometry, binding affinity, or signal strength, among other factors. In the present study, some B*27/57^neg^ LTNP/EC did have alleles, such as B*15, B*44, B*51, B*58 and B*81, that have predisposed individuals to more favorable outcomes (Flores-Villanueva et al., 2001, Frahm et al., 2006, Goulder et al., 2000, Kaslow et al., 1996, Zhang et al., 2011). However, no particular HLA type predominated and approximately 1/3 possessed neutral, deleterious or rare HLA alleles. Taken together these data suggest that the impact of HLA on the development of the LTNP/EC phenotype is not based upon the peptides that are recognized. Although bearing an HLA allele may favor development of the LTNP/EC phenotype, the factors(s) that dictate which patients with a given allele will ultimately restrict virus replication remain open questions. Nevertheless, our results indicate that these factors are highly related to HIV-specific CD8^+^ T-cell cytotoxic capacity.

We did not observe that epitope conservation was a parameter uniquely shared between LTNP/EC with and without protective alleles. An association between lower HIV RNA levels and preferential recognition of conserved epitopes has been suggested in some acute and chronic infection cohorts regardless of HLA type (Dinges et al., 2010, Kunwar et al., 2013, Liu et al., 2009, Liu et al., 2013, Mothe et al., 2011). By contrast, in the present study, the entropies of peptides recognized by the CD8^+^ T-cells of B*27/57^neg^ LTNP/EC and progressors were similar. Responses did tend to exist outside highly variable stretches of the proteome, consistent with some with prior reports (Frahm et al., 2004, Yusim et al., 2002). However, epitopes targeted by B*27/57^neg^ LTNP/EC had the same median entropy as that of epitopes targeted in B*27/57^pos^ LTNP/EC, and the same as that of over 125 well-characterized epitopes restricted by 39 HLA proteins in LANL, the vast majority of which are not associated with reduced viral load. Our results contrast with a very recent study that found an association between immunologic control and targeting of particular epitopes thought to affect HIV protein stability (Pereyra et al., 2014). However, the methods differ from the present study in that these investigators used pre-defined optimal epitopes, rather than mapped responses, and an in silico analysis that did not directly compare targeting among HLA-matched patients with or without control of HIV. In summary, our results suggest that although a highly variable peptide likely cannot mediate control, large numbers of peptides throughout the HIV proteome are sufficiently conserved to mediate potent restriction of HIV replication.

We did find, however, that B*57/27^neg^ LTNP/EC uniformly shared with B*57/27^pos^ LTNP/EC the ability to mediate potent cytotoxicity. In prior work, immunologic control in LTNP/EC was not associated with the frequency of HIV-specific CD8^+^ T cells, surface phenotypic markers, or avidity, among other factors (Hersperger et al., 2011, Migueles et al., 2000, Migueles et al., 2003, Migueles et al., 2008, Migueles et al., 2009). It was also only weakly associated with direct cytotoxicity when using non-restimulated effectors, but was most compellingly associated with CD8^+^ T-cell cytotoxic capacity when measured after 6 days of incubation with autologous HIV-infected targets (Migueles et al., 2008, Migueles et al., 2009). This result is potentially consistent with some prior work suggesting that cytotoxic capacity might be shared between LTNP/EC with and without protective alleles (Hersperger et al., 2010, Lecuroux et al., 2014). The homogeneity in cytotoxic capacity we observed among LTNP/EC may be interpreted to differ with other work that has reported heterogeneity of CD8^+^ T-cell-mediated virus suppression among LTNP/EC, including individuals lacking protective HLA alleles (Lecuroux et al., 2014, Saez-Cirion et al., 2009). These potentially discrepant results may simply be due to differences in experimental conditions used in virus suppression assays compared to assays of cytotoxic capacity. While it remains possible that alternative mechanisms are responsible for controlling HIV in some LTNP/EC, these previous reports and the current study nonetheless suggest that most LTNP/EC with or without protective HLA alleles possess HIV-specific CD8^+^ T-cells with robust virus suppressive and cytotoxic capabilities.

It is important to note that in many cases we observed concordance among protective HLA alleles, immunodominance of responses restricted by those alleles, expansion potential, and cytotoxic capacity. The numerically immunodominant response was often restricted by an HLA previously described as weakly associated with lower viral loads such as *B13*, *15*, *44*, *51*, *58* and *81*. In B*27/57^neg^ LTNP/EC, these same responses efficiently expanded in vitro and mediated potent cytotoxicity. This concordance is consistent with findings in B*27/57^pos^ patients during primary infection, non-progressive chronic infection or following vaccination (Altfeld et al., 2003, Horton et al., 2006, Migueles et al., 2000, Migueles et al., 2002, Migueles et al., 2008, Migueles et al., 2009, Migueles et al., 2011, Saez-Cirion et al., 2007, Saez-Cirion et al., 2009). However, regardless of HLA type, greater cytotoxicity in vitro was not simply due to greater effector numbers, but also due to greater cytotoxic capacity on a per-cell basis. Taken together, we observed that HLAs associated with lower viral loads often restrict immunodominant responses that preferentially expand, upregulate cytotoxic proteins and efficiently lyse HIV-infected CD4^+^ T-cell targets.

Our study has some limitations. A potential shortcoming of our epitope mapping approach was lack of inclusion of variant peptides. Although our results are quite consistent with the comparable recognition of a larger panel of conserved and frequently occurring variant epitopes recently reported between LTNP/EC and progressors (Sunshine et al., 2014), our methods could have potentially biased our results toward detecting responses targeting conserved sequences. In addition, we did not establish cause-and-effect relationships between increased virus-specific CD8^+^ T-cell proliferative and cytotoxic capacities and immunologic control. However, there are several lines of evidence that suggest that cytotoxic capacity is not just an effect of reduced levels of viral replication. CD8^+^ T-cell proliferative and cytotoxic capacities are not restored when HIV RNA levels are suppressed by potent antiretroviral therapy (Migueles et al., 2009). In the rhesus macaque model, infection of animals carrying protective alleles with a cloned, highly pathogenic SIV commonly results in nonprogressive infection and control of SIV replication that is largely removed by CD8^+^ T-cell depletion (Friedrich et al., 2007). While these lines of evidence support that highly functional virus-specific CD8^+^ T cells are not simply an effect of reduced viremia, proof of causality would require control of virus replication following passive transfer of cells with intact proliferative and cytotoxic capacities into humans or animal models.

Finally, the finding that a protective or weakly protective HLA allele is not a requirement to develop high-level restriction of viral replication has some implications for efforts to harness the cellular immune response in vaccines or immunotherapies. Induction of a response capable of mediating immunologic control in diverse populations would therefore not be confined to those expressing HLA molecules capable of presenting critical peptide epitopes. Considering that 25 distinct “weakly” protective or non-protective HLA proteins restricted almost 40 immunodominant CD8^+^ T-cell responses of the B*57/27^neg^ LTNP/EC in the present study, the overall probability of carrying at least one of these alleles in the general population is quite high. Targeting the specificities dictated by HLA is therefore a much lower barrier for the cellular immune response to overcome in the setting of vaccines and immunotherapies than previously thought.

## 5 Author Contributions

D.M., M.G.Z., K.M.M., S.A.T., E.P.K., B.A.P., S.A.J., E.G., K.O.P., H.I. and S.A.M. performed research; D.M., M.G.Z., K.M.M., H.I. and S.A.M. analyzed and interpreted data; H.I. performed virus sequencing; C.W.H. and D.A.F. provided guidance on entropy comparisons and performed statistical analyses; A.P., A.O., C.A.R., S.J. and S.A.M. coordinated inclusion of patients and collected clinical data; C.W.H., S.A.M. and M.C. discussed results; S.A.M., D.M. and M.C. designed the research; D.M., S.A.M. and M.C. wrote the manuscript.

## Figures and Tables

**Table 1:** Dominant CD8^+^ T-cell responses in HLA B*27/57^neg^ patients.

Patients	Epitope	Protein	Frequency	HLA Class I	Epitope/HLA	Patients	Epitope	Protein	Frequency	HLA Class I	Epitope/HLA
LTNP/EC-1	EEMNLPGRW	Pol	0.39	B44	Predicted/predicted	SP-1	KEKGGLEGL	Nef	2.04	B40	Predicted/predicted
HTDNGSNFT^a^	Pol	0.77	C5	Confirmed/confirmed		HTQGYFPDW	Nef	1.82	B58	Confirmed/confirmed
LTNP/EC-2	GSNFTSTTVKAACWW	Pol	0.52				GYFPDWQNY^a^	Nef	2.17	B58	Confirmed/confirmed
LTNP/EC-3	SLYNTVATL	Gag	0.2	A2	Confirmed/predicted		GKKKYKLKHIVWASR	Gag	2.00		
LTNP/EC-4	QVPLRPMTYK	Nef	0.66	A3	Predicted/predicted		LETSEGCRQI^a^	Gag	3.33	B40	Confirmed/confirmed
LTFGWCFKLVPVEPE	Nef	0.69				ILGQLQPSL	Gag	2.20	A2	Predicted/predicted
MARELHPEY	Nef	0.59	B35	Predicted/predicted		ISPRTLNAW	Gag	2.24	B58	Predicted/predicted
RLRPGGKKK	Gag	1.67	A3	Confirmed/predicted		GVGGPGHKARV	Gag	2.36	A2	Confirmed/predicted
NANPDCKTI	Gag	1.18	B51	Confirmed/confirmed		TERQANFL	Gag	1.61	B40	Confirmed/predicted
GVGGPGHK	Gag	0.6	A11	Predicted/predicted	SP-2	TPQDLNTML	Gag	3.99	C8	Confirmed/confirmed
LPPVVAKEI	Pol	0.72	B51	Confirmed/confirmed		SPTRRELQVWGRDNN	Pol	1.37		
LTNP/EC-5	TPQDLNTML	Gag	0.69	B42	Confirmed/confirmed		VTDSQYAL^a^	Pol	1.23	C8	Confirmed/confirmed
LTNP/EC-6	VNDIQKLVGKLNWAS	Pol	0.3			SP-3	KEKGGLEGL	Nef	1	B40	Predicted/predicted
TAYFLLKLAGR	Pol	0.25	A33	Predicted/predicted		GELDRWEKI	Gag	0.5	B40	Predicted/predicted
STTVKAACWW	Pol	0.4	B58	Predicted/predicted		RPLVTIKI	Pol	0.46	B51	Confirmed/confirmed
LTNP/EC-7	RRAEPAADGVGAVSR	Nef	1.18				QWPLTEEKI	Pol	0.79	B51	Predicted/predicted
AAVDLSHFL^a^	Nef	0.7	B58	Confirmed/confirmed		SAGERIVDI	Pol	0.91	B51	Predicted/predicted
VTDSQYAL^a^	Pol	1	C8	Confirmed/confirmed	SP-4	GYFPDWQNY^a^	Nef	0.84	C2	Confirmed/confirmed
KQITKIQNF	Pol	0.77	B58	Predicted/predicted		LTFGWCFKLVPVEPE	Nef	0.9		
LTNP/EC-8	IRYQYNVLP^a^	Pol	0.69	B73	Confirmed/confirmed		RAEQASQEV	Gag	2.56	B51	Predicted/predicted
SPAIFQSSM	Pol	0.5	B35/C4	Predicted/predicted		NANPDCKTI	Gag	1.65	B51	Confirmed/confirmed
HTDNGSNFTSTTVKA	Pol	0.46				VLAEAMSQV	Gag	2.37	A2	Predicted/predicted
GIKQEFGIPYNPQSQ	Pol	0.46				RPLVTIKI	Pol	0.8	B51	Confirmed/confirmed
VVESMNKEL	Pol	0.55	B35	Predicted/predicted		HTDNGSNFT^a^	Pol	2.76	C5	Confirmed/confirmed
RDQAEHLKTAVQMAV	Pol	0.53				IHNFKRKGGIGGYSA	Pol	1.66		
TDIQTKELQKQITKI	Pol	0.57			SP-5	QVPLRPMTYK	Nef	1.24	A3	Predicted/predicted
KIQNFRVYY	Pol	0.5	A32	Predicted/predicted		ETFYVDGAANRETKL	Pol	0.64		
LTNP/EC-10	AAVDLSHFL	Nef	0.55	C3	Confirmed/confirmed		KLAGRWPVK	Pol	0.6	A3	Predicted/predicted
PIVQNLQGQMVHQAI	Gag	0.37			SP-6	SLYNTVATL	Gag	1.33	A2	Confirmed/predicted
LTNP/EC-11	AAVDLSHFL^a^	Nef	0.51	B14	Confirmed/confirmed		SEGATPQDL	Gag	1.92	B44	Confirmed/confirmed
DRFYKTLRA	Gag	1.14	B14	Confirmed/confirmed		EIYKRWII	Gag	1.50	B8	Confirmed/predicted
FKRKGGIGGY	Pol	2.2	B15	Confirmed/predicted	CP-1	SRLAFHHMA^a^	Nef	0.74	B8	Confirmed/confirmed
LTNP/EC-12	WLEAQEEEEVGFPVR	Nef	0.27				HQRIEVKDTKEALEK	Gag	1.32		
RQDILDLW	Nef	0.66	B13	Predicted/predicted		EIYKRWII	Gag	1.59	B8	Confirmed/predicted
LTNP/EC-13	LTFGWCFKL	Nef	1.03	B15	Confirmed/predicted		NANPDCKTI	Gag	3.10	B51	Confirmed/confirmed
LYNTVATLY	Gag	4.17	B44	Confirmed/predicted		RQANFLGK	Gag	1.38	A11	Predicted/predicted
LTNP/EC-14	AVSRDLEK	Nef	2.09	A11	Predicted/predicted		RPLVTIKI	Pol	1.43	B51	Confirmed/confirmed
LTNP/EC-15	DRWEKIRLRPG^a^	Gag	1.45	B40	Confirmed/confirmed		LPPVVAKEI	Pol	1.45	B51	Confirmed/confirmed
MTNNPPIPV	Gag	1.60	C6	Predicted/predicted	CP-2	FPDWQNYTPG	Nef	1.56	B51	Predicted/predicted
LTNP/EC-16	SRLAFHHMA^a^	Nef	1.76	B8	Confirmed/confirmed		EVIPMFSAL	Gag	0.84	B7	Predicted/predicted
YSPTSILDI	Gag	1.61	C1	Confirmed/confirmed		LPPVVAKEI	Pol	0.7	B51	Confirmed/confirmed
LTNP/EC-17	YPLTFGWCF	Nef	1.21	B35	Confirmed/confirmed	CP-3	RPLVTIKI	Pol	1.59	B51	Confirmed/confirmed
NANPDCKTI	Gag	1.52	B35	Confirmed/predicted	CP-4	YPLTFGWCF	Nef	1.22	B51/C14	Predicted/predicted
YRDSRDPLW^a^	Pol	3.24	B38	Confirmed/confirmed		RAEQASQEV	Gag	4.29	B51	Predicted/predicted
LTNP/EC-18	MVHQAISPR	Gag	0.35	A68	Predicted/predicted		NANPDCKTI	Gag	2.00	B51	Confirmed/confirmed
TERQANFL	Gag	0.51	B40	Confirmed/predicted		HTDNGSNFT^a^	Pol	1.17	C5	Confirmed/confirmed
LTNP/EC-19	TPQDLNTML	Gag	1.23	B81	Confirmed/confirmed	CP-5	RERMRRAEPAADGVG	Nef	0.73		
LTNP/EC-20	FLKEKGGL	Nef	0.7	B8	Confirmed/predicted		SEGATPQDL	Gag	2.30	B44	Confirmed/confirmed
LTNP/EC-21	TPQDLNTML	Gag	1.5	B81	Confirmed/confirmed	CP-6	RPQVPLRPM	Nef	1.13	C4	Predicted/predicted
VTDSQYAL^a^	Pol	0.55	C8	Confirmed/confirmed		GLNKIVRMY	Gag	3.64	B15	Predicted/predicted
FKRKGGIGGY	Pol	0.75	B15	Confirmed/confirmed		FIKVRQYDQILIEIC	Pol	0.78		
LTNP/EC-22	TPQDLNTML	Gag	0.3	B42	Confirmed/confirmed		TQIGCTLNF	Pol	0.72	B15	Predicted/predicted
LTNP/EC-23	FLKEKGGL	Nef	1.1	B8	Confirmed/predicted		KQITKIQNF	Pol	0.81	B15	Predicted/predicted
DRFYKTLRA	Gag	0.57	B14	Confirmed/confirmed	CP-7	FLKEKGGL	Nef	1.28	A2	Predicted/predicted
							NETPGIRYQY	Pol	0.86	B18	Predicted/predicted
						FYVDGAANR	Pol	1.18	A33	Predicted/predicted

a Epitope-specific responses not previously reported.

**Table 2:** Confirmed (by epitope) dominant CD8^+^ T-cell responses by HLA restriction element.

HLA class I protein	Epitope	HIV-1 protein	Recognition frequency^a^	Dominant response frequency^b^	Patients with dominant response	HLA
A2	SLYNTVATL	Gag	5/14	2/5	LTNP/EC-3, SP-6	Predicted
A2	GVGGPGHKARV	Gag	2/14	1/2	SP-1	Predicted
A3	RLRPGGKKK	Gag	1/5	1/1	LTNP/EC-4	Predicted
B8	FLKEKGGL	Nef	3/5	2/3	LTNP/EC-20, LTNP/EC-23	Predicted
B8	SRLAFHHMA^c^	Nef	2/5	1/2	LTNP/EC-16, CP-1	Confirmed
B8	EIYKRWII	Gag	4/5	2/4	SP-6, CP-1	Predicted
B14	DRFYKTLRA	Gag	3/3	2/3	LTNP/EC-11, LTNP/EC-23	Confirmed
B15	LTFGWCFKL	Nef	2/8	1/2	LTNP/EC-13	Predicted
B15	FKRKGGIGGY	Pol	4/8	2/4	LTNP/EC-11, LTNP/EC-21	Confirmed
B35	YPLTFGWCF	Nef	2/4	1/2	LTNP/EC-17	Confirmed
B38	YRDSRDPLW^c^	Pol	1/1	1/1	LTNP/EC-17	Confirmed
B40	DRWEKIRLRPG^c^	Gag	3/4	1/3	LTNP/EC-15	Confirmed
B40	LETSEGCRQI^c^	Gag	1/4	1/1	SP-1	Confirmed
B40	TERQANFL	Gag	3/4	2/3	LTNP/EC-18, SP-1	Predicted
B44	LYNTVATLY	Gag	2/10	1/2	LTNP/EC-13	Predicted
B44	SEGATPQDL	Gag	2/10	2/2	SP-6, CP-5	Confirmed
B51	RPLVTIKI	Pol	4/8	4/4	SP-3, SP-4, CP-1, CP-3	Confirmed
B51	LPPVVAKEI	Pol	7/8	3/7	LTNP/EC-4, CP-1, CP-2	Confirmed
B58	HTQGYFPDW	Nef	1/4	1/1	SP-1	Confirmed
B73	IRYQYNVLP^c^	Pol	1/1	1/1	LTNP/EC-8	Confirmed
C1	YSPTSILDI	Gag	1/1	1/1	LTNP/EC-16	Confirmed
C5	HTDNGSNFT^c^	Pol	3/5	3/3	LTNP/EC-1, SP-4, CP-4	Confirmed
C8	VTDSQYAL^c^	Pol	3/5	3/3	LTNP/EC-7, LTNP/EC-21, SP-2	Confirmed
B14	AAVDLSHFL^c^	Nef	1/3	1/1	LTNP/EC-11	Confirmed
B58	AAVDLSHFL^c^	Nef	1/4	1/1	LTNP/EC-7	Confirmed
C3	AAVDLSHFL	Nef	3/7	1/3	LTNP/EC-10	Confirmed
B58	GYFPDWQNY^c^	Nef	1/4	1/1	SP-1	Confirmed
C2	GYFPDWQNY^c^	Nef	2/7	1/2	SP-4	Confirmed
B42	TPQDLNTML	Gag	2/2	2/2	LTNP/EC-5, LTNP/EC-22	Confirmed
B81	TPQDLNTML	Gag	2/2	2/2	LTNP/EC-19, LTNP/EC-21	Confirmed
C8	TPQDLNTML	Gag	2/5	1/2	SP-2	Confirmed
B35	NANPDCKTI	Gag	2/4	1/2	LTNP/EC-17	Predicted
B51	NANPDCKTI	Gag	4/8	3/4	LTNP/EC-4, SP-4, CP-1, CP-4	Confirmed

a Number of individuals recognizing the 15-mer peptide and/or optimal epitope out of those with relevant HLA type.

b Number of individuals with a dominant response to the peptide out of those recognizing the peptide.

c Epitope-specific responses not previously reported.

**Table 3:**
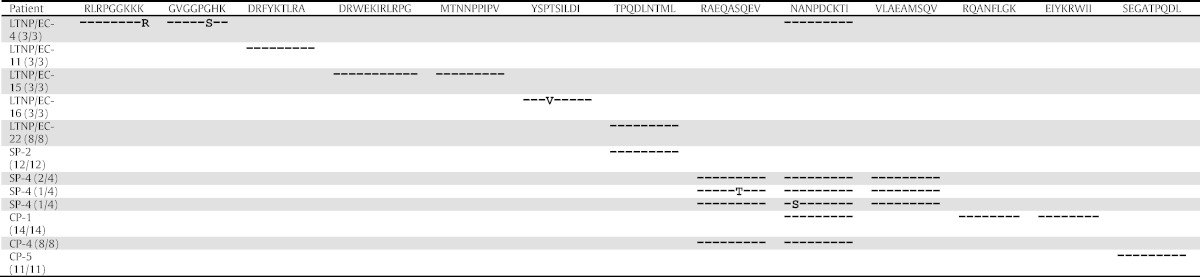
Gag epitope sequences in B*27/57^neg^ patients.
