# Supplementary material for: CD8+ T-cell Cytotoxic Capacity Associated with Human Immunodeficiency Virus-1 Control Can Be Mediated through Various Epitopes and Human Leukocyte Antigen Types
Source: eBioMedicine. 2014 Dec 22;2(1):46–58. doi: 10.1016/j.ebiom.2014.12.009 (PMC4485486; doi:10.1016/j.ebiom.2014.12.009)
Supplement: Supplementary file 1 — Supplementary tables. [file mmc1.docx]

**Table S1.** Patient characteristics

| **Patient** | **Diagnosis** | **Gender** | **Race/** | **Mode** | **HLA-A** | **HLA-B** | **HLA-C** | **CD4 Count** | **VL** |
| --- | --- | --- | --- | --- | --- | --- | --- | --- | --- |
|  | **Year** |  | **Ethnicity** |  |  |  |  | **(Cells/mm^3^)** | **(Copies/mL)** |
| ***B*27/57^neg^* LTNP/EC** | |  |  |  |  |  |  |  |  |
| LTNP/EC-1 | 2007 | M | Caucasian | MSM | 24,29 | 44 | 5,16 | 740 | <50 |
| LTNP/EC-2 | 2003 | M | Caucasian | MSM | 2,31 | 44, 51 | 5,15 | 362 | <50 |
| LTNP/EC-3 | 2004 | M | Caucasian | MSM | 2,29 | 44, 49 | 7,16 | 663 | <50 |
| LTNP/EC-4 | 1981 | M | Caucasian | MSM | 3,11 | 35, 51 | 4,15 | 967 | <50 |
| LTNP/EC-5 | 1998 | F | AA | Heterosexual | 30,33 | 42, 58 | 2,17 | 1813 | <50-59 |
| LTNP/EC-6 | 2004 | F | AA | Heterosexual | 23,33 | 58 | 6 | 522 | <50 |
| LTNP/EC-7 | 1989 | M | AA | MSM | 29,32 | 14, 58 | 3,8 | 1209 | <50 |
| LTNP/EC-8 | 1985 | M | Caucasian | MSM | 1,32 | 35, 73 | 4,15 | 1060 | <50 |
| LTNP/EC-9 | 1988 | M | Caucasian | MSM | 11,32 | 35, 50 | 4,6 | 665 | <50-71 |
| LTNP/EC-10 | 2001 | M | Caucasian | MSM | 11,30 | 15,52 | 3,12 | 464 | <50 |
| LTNP/EC-11 | 2005 | F | AA | Heterosexual | 33,68 | 14,15 | 2,8 | 651 | <50-325 |
| LTNP/EC-12 | 1996 | M | Caucasian | IVDU | 29,30 | 7,13 | 6,15 | 1284 | <50 |
| LTNP/EC-13 | 2004 | F | Hispanic | Heterosexual | 3,24 | 15,44 | 7,14 | 1360 | <50-168 |
| LTNP/EC-14 | 1985 | M | Caucasian | MSM | 1,11 | 35,47 | 4,6 | 682 | <50 |
| LTNP/EC-15 | 1985 | M | Caucasian | MSM | 30,32 | 13,40 | 3,6 | 925 | <50-81 |
| LTNP/EC-16 | 1989 | M | AA | MSM | 1,33 | 8,53 | 1,4 | 913 | <50 |
| LTNP/EC-17 | 1982 | M | Caucasian | MSM | 11,24 | 35,38 | 4,12 | 537 | <50 |
| LTNP/EC-18 | 1992 | M | Caucasian | MSM | 3,68 | 40,53 | 2,4 | 1084 | <50 |
| LTNP/EC-19 | 2007 | F | AA | Heterosexual | 3,30 | 53,81 | 4,18 | 1943 | <50 |
| LTNP/EC-20 | 2003 | M | Caucasian | MSM | 2 | 8,49 | 7 | 774 | <50 |
| LTNP/EC-21 | 2000 | F | AA | Heterosexual | 1,2 | 15,81 | 8,18 | 964 | <50 |
| LTNP/EC-22 | 1987 | T | AA | MSM | 2,33 | 15,42 | 14,17 | 570 | <50 |
| LTNP/EC-23 | 1982 | M | Caucasian | MSM | 1,33 | 8,14 | 7,8 | 1211 | <50 |
| **Slow Progressors** | |  |  |  |  |  |  |  |  |
| SP1 | 1982 | M | Caucasian | Hemophiliac | 2,3 | 40, 58 | 3,7 | 1006 | 2599 |
| SP2 | 1991 | F | AA | Heterosexual | 23,30 | 15,41 | 2,8 | 478 | 6343 |
| SP3 | 2004 | M | Hispanic | MSM | 24 | 40,51 | 3,15 | 550 | 1470 |
| SP4 | 1996 | M | Caucasian | MSM | 2,30 | 44,51 | 2,5 | 720 | 1214 |
| SP5 | 1995 | F | AA | Heterosexual | 03,66 | 44,49 | 4,7 | 461 | 2059 |
| SP6 | 2003 | M | Caucasian | MSM | 2,68 | 8, 44 | 2,7 | 563 | 1922 |
| **Progressors** | |  |  |  |  |  |  |  |  |
| CP1 | 2001 | M | Caucasian | MSM | 2,11 | 8,51 | 4,7 | 294 | 15129 |
| CP2 | 1985 | M | Caucasian | MSM | 2 | 07,51 | 7,14 | 312 | 10443 |
| CP3 | 1997 | M | AA | Heterosexual | 2,68 | 44, 51 | 5,16 | 422 | 18220 |
| CP4 | 2000 | M | Caucasian | MSM | 2 | 44, 51 | 5,14 | 304 | 29417 |
| CP5 | 1990 | M | Caucasian | MSM | 1,24 | 7, 44 | 7,16 | 185 | 83390 |
| CP6 | 1985 | M | Caucasian | MSM | 2 | 15 | 3,4 | 571 | 20969 |
| CP7 | 1994 | M | AA | MSM | 2,33 | 15,18 | 2,14 | 351 | 62949 |
| ***B*27/57^pos^* LTNP/EC** | |  |  |  |  |  |  |  |  |
| B*27/57+ 1 | 1985 | M | Caucasian | MSM | 1,31 | 8,57 | 6,7 | 1060 | <50 |
| B*27/57+ 2 | 1985 | F | Caucasian | Transfusion | 1,2 | 57 | 6 | 474 | <50-172 |
| B*27/57+ 3 | 1984 | M | Caucasian | MSM | 11,23 | 44, 57 | 4,6 | 590 | <50-930 |
| B*27/57+ 4 | 1985 | M | Caucasian | MSM | 2,26 | 27,38 | 1,12 | 964 | <50 |
| B*27/57+ 5 | 1989 | F | AA | Heterosexual | 30,74 | 15,57 | 3,8 | 602 | <50 |
| B*27/57+ 6 | 1982 | F | Caucasian | Transfusion | 32 | 27,44 | 1,5 | 1247 | <50-201 |
| B*27/57+ 7 | 1996 | M | Caucasian | Heterosexual | 1,24 | 38,57 | 6,12 | 1076 | <50 |
| B*27/57+ 8 | 1991 | M | Caucasian | MSM | 2,3 | 7,57 | 6,7 | 753 | <50 |
| B*27/57+ 9 | 1998 | M | AA | Heterosexual | 1,30 | 42,57 | 7,17 | 684 | <50 |
| B*27/57+ 10 | 1987 | M | Caucasian | MSM | 1,2 | 37,57 | 6 | 566 | <50-304 |
| B*27/57+ 11 | 2001 | M | Caucasian | MSM | 1,29 | 52,57 | 6,12 | 777 | <50 |
| B*27/57+ 12 | 1978 | M | Caucasian | MSM | 3,26 | 14,57 | 7 | 685 | <50 |
| B*27/57+ 13 | 1993 | M | Caucasian | MSM | 1,3 | 8,57 | 6,7 | 1208 | <50 |
| B*27/57+ 14 | 1985 | M | Caucasian | MSM | 1,25 | 18,57 | 6,12 | 679 | <50 |
| B*27/57+ 15 | 1991 | M | AA | MSM | 24,32 | 27,81 | 2,8 | 693 | <50 |
| B*27/57+ 16 | 1989 | M | Caucasian | MSM | 26,31 | 27,38 | 2,12 | 854 | <50-340 |
| B*27/57+ 17 | 1988 | M | Caucasian | MSM | 1,2 | 51,57 | 6,14 | 649 | <50 |
| B*27/57+ 18 | 2006 | F | AA | Heterosexual | 30,33 | 7,57 | 7,8 | 1259 | <50-203 |
| B*27/57+ 19 | 1987 | F | Caucasian | Heterosexual | 1,26 | 27,57 | 2,6 | 378 | <50 |
| B*27/57+ 20 | 2003 | F | Hispanic | Heterosexual | 1 | 15,57 | 3,6 | 1311 | <50 |
| B*27/57+ 21 | 2004 | M | AA | MSM | 2,31 | 15,57 | 3,7 | 1263 | <50 |
| B*27/57+ 22 | 1999 | F | AA | Heterosexual | 1,2 | 8,27 | 1,7 | 941 | <50 |
| B*27/57+ 23 | 1985 | M | Hispanic | IVDU | 29,68 | 44,57 | 7,16 | 716 | <50 |

LTNP/EC, long-term nonprogressor / elite controller; M, male; F, female; T, transgendered (male to female); AA, African American; MSM, men who have sex with men; HLA, human leukocyte antigen; VL, viral load

**Table S2.** LTNP/EC 15-mer Peptide Response Summary

| **Patients** | **Nef Peptides** | **Nef Frequency** | **Gag Peptides** | **Gag Frequency** | **Pol Peptides** | **Pol Frequency** |
| --- | --- | --- | --- | --- | --- | --- |
| **LTNP/EC-1** | **IRYPLTFGWCFKLVP** | **0.24** | **RAEQASQEVKNWMTE** | **0.20** | **LEEMNLPGRWKPKMI** | **0.39** |
|  |  |  |  |  | **IEELRQHLLRWGFTT** | **0.07** |
|  |  |  |  |  | **VKTIHTDNGSNFTST** | **0.69** |
| **LTNP/EC-2** |  |  | **SLYNTVATLYCVHQR** | **0.15** | **EEHEKYHSNWRAMAS** | **0.25** |
|  |  |  |  |  | **GSNFTSTTVKAACWW** | **0.52** |
| **LTNP/EC-3** |  |  | **SLYNTVATLYCVHQR** | **0.2** |  |  |
| **LTNP/EC-4** | **SSNTAANNADCAWLE** | **0.17** | **EKIRLRPGGKKKYKL** | **1.67** | **AIFQSSMTKILEPFR** | **0.21** |
|  | **ADCAWLEAQEEEEVG** | **0.32** | **NANPDCKTILKALGP** | **0.33** | **QNPDIVIYQYMDDLY** | **0.30** |
|  | **QEEEEVGFPVRPQVP** | **0.47** | **ACQGVGGPGHKARVL** | **0.6** | **TYQIYQEPFKNLKTG** | **0.45** |
|  | **QVPLRPMTYKAAVDL** | **0.66** |  |  | **MASDFNLPPVVAKEI** | **0.72** |
|  | **PGPGIRYPLTFGWCF** | **0.46** |  |  |  |  |
|  | **LTFGWCFKLVPVEPE** | **0.69** |  |  |  |  |
|  | **LVPVEPEKVEEANEG** | **0.55** |  |  |  |  |
|  | **NNSLLHPMSLHGMDD** | **0.55** |  |  |  |  |
|  | **SLHGMDDPEREVLEW** | **0.35** |  |  |  |  |
|  | **EREVLEWKFDSRLAF** | **0.20** |  |  |  |  |
|  | **FDSRLAFHHMARELH** | **0.35** |  |  |  |  |
|  | **HMARELHPEYYKDC** | **0.59** |  |  |  |  |
| **LTNP/EC-5** |  |  | **EGATPQDLNTMLNTV** | **0.88** | **QKQGQGQWTYQIYQE** | **0.21** |
| **LTNP/EC-6** |  |  | **PVHAGPIAPGQMREP** | **0.12** | **KWTVQPIVLPEKDSW** | **0.19** |
|  |  |  | **PGQMREPRGSDIAGT** | **0.12** | **VNDIQKLVGKLNWAS** | **0.3** |
|  |  |  |  |  | **GQETAYFLLKLAGRW** | **0.25** |
|  |  |  |  |  | **TSTTVKAACWWAGIK** | **0.4** |
|  |  |  |  |  | **MNKELKKIIGQVRDQ** | **0.1** |
| **LTNP/EC-7** | **RRAEPAADGVGAVSR** | **1.18** | **SGGELDRWEKIRLRP** | **0.17** | **IRYQYNVLPQGWKGS** | **0.22** |
|  | **YKAAVDLSHFLKEKG** | **0.71** | **GKKKYKLKHIVWASR** | **0.3** | **GLEVNIVTDSQYALG** | **1** |
|  |  |  | **EELRSLYNTVATLYC** | **0.42** | **QKQITKIQNFRVYYR** | **0.77** |
|  |  |  | **EGATPQDLNTMLNTV** | **0.32** |  |  |
|  |  |  | **LKETINEEAAEWDRL** | **0.21** |  |  |
|  |  |  | **VDRFYKTLRAEQASQ** | **0.17** |  |  |
|  |  |  | **RAEQASQEVKNWMTE** | **0.16** |  |  |
| **LTNP/EC-8** |  |  |  |  | **ETPGIRYQYNVLPQG** | **0.69** |
|  |  |  |  |  | **YNVLPQGWKGSPAIF** | **0.35** |
|  |  |  |  |  | **KGSPAIFQSSMTKIL** | **0.5** |
|  |  |  |  |  | **PFRKQNPDIVIYQYM** | **0.29** |
|  |  |  |  |  | **IVIYQYMDDLYVGSD** | **0.19** |
|  |  |  |  |  | **LVAVHVASGYIEAEV** | **0.25** |
|  |  |  |  |  | **GYIEAEVIPAETGQE** | **0.35** |
|  |  |  |  |  | **PAETGQETAYFLLKL** | **0.34** |
|  |  |  |  |  | **LKLAGRWPVKTIHTD** | **0.42** |
|  |  |  |  |  | **HTDNGSNFTSTTVKA** | **0.46** |
|  |  |  |  |  | **VKAACWWAGIKQEFG** | **0.26** |
|  |  |  |  |  | **GIKQEFGIPYNPQSQ** | **0.46** |
|  |  |  |  |  | **QSQGVVESMNKELKK** | **0.55** |
|  |  |  |  |  | **LKKIIGQVRDQAEHL** | **0.32** |
|  |  |  |  |  | **RDQAEHLKTAVQMAV** | **0.53** |
|  |  |  |  |  | **TAVQMAVFIHNFKRK** | **0.25** |
|  |  |  |  |  | **KRKGGIGGYSAGERI** | **0.42** |
|  |  |  |  |  | **ERIVDIIATDIQTKE** | **0.3** |
|  |  |  |  |  | **TDIQTKELQKQITKI** | **0.57** |
|  |  |  |  |  | **QKQITKIQNFRVYYR** | **0.5** |
|  |  |  |  |  | **NFRVYYRDSRDPLWK** | **0.29** |
| **LTNP/EC-10** | **RPMTYKAAVDLSHFL** | **0.47** | **PIVQNLQGQMVHQAI** | **0.37** |  |  |
|  | **VYHTQGYFPDWQNY** | **0.14** |  |  |  |  |
| **LTNP/EC-11** | **RPMTYKAAVDLSHFL** | **0.45** | **FRDYVDRFYKTLRAE** | **1.62** | **FIKVRQYDQILIEIC** | **0.24** |
|  |  |  | **KEGHIAKNCRAPRKK** | **0.20** | **IGRNLLTQIGCTLNF** | **0.69** |
|  |  |  | **KELYPLASLRSLFGN** | **0.15** | **IHNFKRKGGIGGYSA** | **2.17** |
| **LTNP/EC-12** | **SSNTAANNADCAWLE** | **0.2** |  |  | **SINNETPGIRYQYNV** | **0.16** |
|  | **WLEAQEEEEVGFPVR** | **0.27** |  |  | **KGSPAIFQSSMTKIL** | **0.09** |
|  | **EVGFPVRPQVPLRPM** | **0.26** |  |  |  |  |
|  | **QVPLRPMTYKAAVDL** | **0.15** |  |  |  |  |
|  | **IYSQKRQDILDLWVY** | **0.66** |  |  |  |  |
| **LTNP/EC-13** | **FPDWQNYTPGPGIRY** | **0.14** | **EELRSLYNTVATLYC** | **4.42** |  |  |
|  | **LTFGWCFKLVPVEPE** | **0.41** | **HQRIEVKDTKEALEK** | **0.16** |  |  |
|  |  |  | **FRDYVDRFYKTLRAE** | **0.17** |  |  |
|  |  |  | **RAEQASQEVKNWMTE** | **0.68** |  |  |
|  |  |  | **NANPDCKTILKALGP** | **0.24** |  |  |
| **LTNP/EC-14** | **WPTVRERMRRAEPAA** | **0.24** |  |  |  |  |
|  | **RRAEPAADGVGAVSR** | **0.12** |  |  |  |  |
|  | **GVGAVSRDLEKHGAI** | **2.09** |  |  |  |  |
|  | **ADCAWLEAQEEEEVG** | **0.31** |  |  |  |  |
|  | **PVRPQVPLRPMTYKA** | **0.58** |  |  |  |  |
| **LTNP/EC-15** |  |  | **LDRWEKIRLRPGGKK** | **1.56** |  |  |
|  |  |  | **WMTNNPPIPVGEIYK** | **1.60** |  |  |
| **LTNP/EC-16** | **PGPGIRYPLTFGWCF** | **0.36** | **EKAFSPEVIPMFSAL** | **0.17** |  |  |
|  | **FDSRLAFHHMARELH** | **0.58** | **IPMFSALSEGATPQD** | **0.29** |  |  |
|  |  |  | **YSPTSILDIRQGPKE** | **0.75** |  |  |
| **LTNP/EC-17** | **QEEEEVGFPVRPQVP** | **0.65** | **LLVQNANPDCKTILK** | **1.52** | **NFRVYYRDSRDPLWK** | **2.56** |
|  | **PGPGIRYPLTFGWCF** | **1.13** |  |  |  |  |
| **LTNP/EC-18** |  |  | **QMVHQAISPRTLNAW** | **0.35** |  |  |
|  |  |  | **INEEAAEWDRLHPVH** | **0.2** |  |  |
|  |  |  | **DRLHPVHAGPIAPGQ** | **0.28** |  |  |
|  |  |  | **DCTERQANFLGKIWP** | **0.39** |  |  |
| **LTNP/EC-19** |  |  | **EGATPQDLNTMLNTV** | **0.58** |  |  |
|  |  |  | **FNCGKEGHIAKNCRA** | **0.28** |  |  |
| **LTNP/EC-20** | **VDLSHFLKEKGGLEG** | **0.65** | **NPPIPVGEIYKRWII** | **0.13** |  |  |
| **LTNP/EC-21** | **PVRPQVPLRPMTYKA** | **0.5** | **EGATPQDLNTMLNTV** | **1.50** | **NIVTDSQYALGIIQA** | **0.55** |
|  | **YKAAVDLSHFLKEKG** | **0.32** |  |  | **IHNFKRKGGIGGYSA** | **0.11** |
| **LTNP/EC-22** | **TQGYFPDWQNYTPGP** | **0.12** | **EGATPQDLNTMLNTV** | **0.30** |  |  |
| **LTNP/EC-23** | **VDLSHFLKEKGGLEG** | **1.09** | **NLQGQMVHQAISPRT** | **0.32** | **VIQDNSDIKVVPRRK** | **0.17** |
|  | **IYSQKRQDILDLWVY** | **0.16** | **NPPIPVGEIYKRWII** | **0.28** |  |  |
|  |  |  | **YSPTSILDIRQGPKE** | **0.13** |  |  |
|  |  |  | **FRDYVDRFYKTLRAE** | **0.60** |  |  |

**Table S3.** Slow Progressor 15-mer Peptide Response Summary

| **Patients** | **Nef Peptides** | **Nef Frequency** | **Gag Peptides** | **Gag Frequency** | **Pol Peptides** | **Pol Frequency** |
| --- | --- | --- | --- | --- | --- | --- |
| **SP-1** | **QVPLRPMTYKAAVDL** | **0.29** | **MGARASVLSGGELDR** | **0.15** | **RQYDQILIEICGHKA** | **0.36** |
|  | **HFLKEKGGLEGLIYS** | **2.04** | **LDRWEKIRLRPGGKK** | **0.43** | **GTVLVGPTPVNIIGR** | **0.62** |
|  | **VYHTQGYFPDWQNY** | **1.43** | **GKKKYKLKHIVWASR** | **2.00** | **ISPIETVPVKLKPGM** | **1.31** |
|  | **LTFGWCFKLVPVEPE** | **0.62** | **GLLETSEGCRQILGQ** | **2.36** | **KQWPLTEEKIKALVE** | **0.23** |
|  | **EREVLEWKFDSRLAF** | **0.32** | **CRQILGQLQPSLQTG** | **2.20** | **IVIYQYMDDLYVGSD** | **0.43** |
|  |  |  | **HQRIEVKDTKEALEK** | **0.12** | **HRTKIEELRQHLLRW** | **0.61** |
|  |  |  | **EEEQNKSKKKAQQAA** | **0.25** | **IEELRQHLLRWGFTT** | **0.37** |
|  |  |  | **NLQGQMVHQAISPRT** | **1.68** | **QPIVLPEKDSWTVND** | **0.27** |
|  |  |  | **QAISPRTLNAWVKVV** | **2.24** | **VNDIQKLVGKLNWAS** | **0.22** |
|  |  |  | **EKAFSPEVIPMFSAL** | **0.85** | **TEVIPLTEEAELELA** | **0.41** |
|  |  |  | **ASQEVKNWMTETLLV** | **0.65** | **EAVQKIATESIVIWG** | **1.29** |
|  |  |  | **ACQGVGGPGHKARVL** | **2.20** | **DSQYALGIIQAQPDK** | **0.64** |
|  |  |  | **RVLAEAMSQVTNSAT** | **0.81** | **ESELVSQIIEQLIKK** | **0.57** |
|  |  |  | **SATIMMQRGNFRNQR** | **0.71** | **IEQLIKKEKVYLAVVV** | **0.4** |
|  |  |  | **HQMKDCTERQANFLG** | **1.61** | **LVAVHVASGYIEAEV** | **0.29** |
|  |  |  |  |  | **LKLAGRWPVKTIHTD** | **0.97** |
|  |  |  |  |  | **VVESMNKELKKIIGQ** | **0.42** |
|  |  |  |  |  | **LKKIIGQVRDQAEHL** | **0.39** |
| **SP-2** | **IRYPLTFGWCFKLVP** | **0.31** | **GKKKYKLKHIVWASR** | **0.60** | **AREFSSEQTRANSPT** | **0.69** |
|  |  |  | **EELRSLYNTVATLYC** | **0.31** | **SPTRRELQVWGRDNN** | **1.37** |
|  |  |  | **EGATPQDLNTMLNTV** | **3.80** | **VWGRDNNSLSEAGAD** | **0.73** |
|  |  |  |  |  | **DNNSLSEAGADRQGT** | **0.57** |
|  |  |  |  |  | **GKLNWASQIYAGIKV** | **1.17** |
|  |  |  |  |  | **GLEVNIVTDSQYALG** | **1.23** |
|  |  |  |  |  | **IHNFKRKGGIGGYSA** | **0.44** |
|  |  |  |  |  | **NFRVYYRDSRDPLWK** | **0.31** |
| **SP-3** | **HFLKEKGGLEGLIYS** | **1** | **ASVLSGGELDRWEKI** | **0.5** | **LWQRPLVTIKIGGQL** | **0.46** |
|  | **ILDLWVYHTQGYFPD** | **0.12** | **YKLKHIVWASRELER** | **0.09** | **ISPIETVPVKLKPGM** | **0.22** |
|  | **TQGYFPDWQNYTPGP** | **0.11** | **LKETINEEAAEWDRL** | **0.21** | **GPKVKQWPLTEEKIK** | **0.79** |
|  | **QNYTPGPGIRYPLTF** | **0.19** | **AAEWDRLHPVHAGPI** | **0.08** | **TDTTNQKTELQAIHL** | **0.32** |
|  | **WCFKLVPVEPEKVEE** | **0.19** | **DCTERQANFLGKIWP** | **0.42** | **FNLPPVVAKEIVASC** | **0.39** |
|  | **NEGENNSLLHPMSLH** | **0.43** |  |  | **YSAGERIVDIIATDI** | **0.91** |
|  |  |  |  |  | **TKELQKQITKIQNFR** | **0.44** |
| **SP-4** | **RERMRRAEPAADGVG** | **0.11** | **RAEQASQEVKNWMTE** | **2.56** | **AREFSSEQTRANSPT** | **0.72** |
|  | **TQGYFPDWQNYTPGP** | **0.72** | **LLVQNANPDCKTILK** | **0.47** | **SPTRRELQVWGRDNN** | **0.64** |
|  | **QNYTPGPGIRYPLTF** | **0.45** | **ATLEEMMTACQGVGG** | **0.27** | **VWGRDNNSLSEAGAD** | **0.45** |
|  | **LTFGWCFKLVPVEPE** | **0.9** | **ACQGVGGPGHKARVL** | **0.25** | **QGTVSFSFPQITLWQ** | **0.53** |
|  | **NEGENNSLLHPMSLH** | **0.39** | **GHKARVLAEAMSQVT** | **2.37** | **LWQRPLVTIKIGGQL** | **0.79** |
|  |  |  | **QVTNSATIMMQRGNF** | **0.34** | **GKLNWASQIYAGIKV** | **0.86** |
|  |  |  | **MMQRGNFRNQRKTVK** | **0.22** | **LLRGTKALTEVIPLT** | **0.42** |
|  |  |  | **TVKCFNCGKEGHIAK** | **0.27** | **YARMRGAHTNDVKQL** | **0.27** |
|  |  |  | **RKKGCWKCGKEGHQM** | **0.23** | **KQLTEAVQKIATESI** | **0.58** |
|  |  |  | **HQMKDCTERQANFLG** | **0.18** | **KIATESIVIWGKTPK** | **0.14** |
|  |  |  | **RQANFLGKIWPSHKG** | **0.76** | **TPKFKLPIQKETWEA** | **0.22** |
|  |  |  | **IWPSHKGRPGNFLQS** | **0.34** | **IEQLIKKEKVYLAVVV** | **0.4** |
|  |  |  | **KELYPLASLRSLFGN** | **0.22** | **MASDFNLPPVVAKEI** | **0.23** |
|  |  |  |  |  | **KCQLKGEAMHGQVDC** | **0.28** |
|  |  |  |  |  | **VKTIHTDNGSNFTST** | **2.76** |
|  |  |  |  |  | **IHNFKRKGGIGGYSA** | **1.66** |
|  |  |  |  |  | **YSAGERIVDIIATDI** | **0.4** |
|  |  |  |  |  | **TKELQKQITKIQNFR** | **0.2** |
|  |  |  |  |  | **PAKLLWKGEGAVVIQ** | **0.75** |
| **SP-5** | **WLEAQEEEEVGFPVR** | **0.13** |  |  | **LVKLWYQLEKEPIVG** | **0.41** |
|  | **QVPLRPMTYKAAVDL** | **1.24** |  |  | **ETFYVDGAANRETKL** | **0.64** |
|  |  |  |  |  | **ANRETKLGKAGYVTD** | **0.26** |
|  |  |  |  |  | **GRQKVVSLTDTTNQK** | **0.36** |
|  |  |  |  |  | **TDTTNQKTELQAIHL** | **0.21** |
|  |  |  |  |  | **GQETAYFLLKLAGRW** | **0.34** |
|  |  |  |  |  | **LKLAGRWPVKTIHTD** | **0.6** |
|  |  |  |  |  | **TSTTVKAACWWAGIK** | **0.5** |
| **SP-6** | **VDLSHFLKEKGGLEG** | **0.26** | **SLYNTVATLYCVHQR** | **1.33** |  |  |
|  | **IYSQKRQDILDLWVY** | **0.22** | **HQRIEVKDTKEALEK** | **0.50** |  |  |
|  | **IRYPLTFGWCFKLVP** | **0.1** | **SALSEGATPQDLNTM** | **2.60** |  |  |
|  | **VEEANEGENNSLLHP** | **0.34** | **LKETINEEAAEWDRL** | **0.53** |  |  |
|  |  |  | **PVHAGPIAPGQMREP** | **0.60** |  |  |
|  |  |  | **PGQMREPRGSDIAGT** | **0.78** |  |  |
|  |  |  | **AGTTSTLQEQIGWMT** | **0.29** |  |  |
|  |  |  | **NPPIPVGEIYKRWII** | **1.50** |  |  |
|  |  |  | **RAEQASQEVKNWMTE** | **0.23** |  |  |
|  |  |  | **LLVQNANPDCKTILK** | **0.42** |  |  |

**Table S4.** Chronic Progressor 15-mer Peptide Response Summary

| **Patients** | **Nef Peptides** | **Nef Frequency** | **Gag Peptides** | **Gag Frequency** | **Pol Peptides** | **Pol Frequency** |
| --- | --- | --- | --- | --- | --- | --- |
| **CP-1** | **QVPLRPMTYKAAVDL** | **0.68** | **SLYNTVATLYCVHQR** | **0.21** | **LWQRPLVTIKIGGQL** | **2.07** |
|  | **YKAAVDLSHFLKEKG** | **0.43** | **HQRIEVKDTKEALEK** | **1.32** | **VQLGIPHPAGLKKKK** | **1.13** |
|  | **KRQDILDLWVYHTQG** | **0.22** | **NPPIPVGEIYKRWII** | **1.59** | **KYTAFTIPSINNETP** | **0.61** |
|  | **TQGYFPDWQNYTPGP** | **0.15** | **YSPTSILDIRQGPKE** | **0.22** | **KGSPAIFQSSMTKIL** | **0.53** |
|  | **LEWKFDSRLAFHHMA** | **0.77** | **LLVQNANPDCKTILK** | **3.39** | **PFRKQNPDIVIYQYM** | **0.22** |
|  |  |  | **DCTERQANFLGKIWP** | **1.38** | **WASQIYAGIKVKQLC** | **0.66** |
|  |  |  | **FLGKIWPSHKGRPGN** | **1.30** | **LQDSGLEVNIVTDSQ** | **0.12** |
|  |  |  |  |  | **ESELVSQIIEQLIKK** | **0.27** |
|  |  |  |  |  | **MASDFNLPPVVAKEI** | **0.38** |
| **CP-2** | **FPDWQNYTPGPGIRY** | **1.56** | **QAISPRTLNAWVKVV** | **0.32** | **FNLPPVVAKEIVASC** | **0.75** |
|  | **IRYPLTFGWCFKLVP** | **0.29** | **EKAFSPEVIPMFSAL** | **0.84** |  |  |
|  | **LVPVEPEKVEEANEG** | **0.28** | **EGATPQDLNTMLNTV** | **0.44** |  |  |
| **CP-3** | **IRYPLTFGWCFKLVP** | **0.21** | **GLLETSEGCRQILGQ** | **0.30** | **PQITLWQRPLVTIKI** | **1.59** |
|  | **VEEANEGENNSLLHP** | **0.16** | **EELRSLYNTVATLYC** | **0.36** | **RELNKRTQDFWEVQL** | **0.1** |
|  |  |  |  |  | **MASDFNLPPVVAKEI** | **0.2** |
| **CP-4** | **FPDWQNYTPGPGIRY** | **0.25** | **RAEQASQEVKNWMTE** | **4.29** | **DDTVLEEMNLPGRWK** | **0.3** |
|  | **IRYPLTFGWCFKLVP** | **1.22** | **NANPDCKTILKALGP** | **2.00** | **HRTKIEELRQHLLRW** | **0.17** |
|  | **LVPVEPEKVEEANEG** | **0.28** | **TVKCFNCGKEGHIAK** | **0.83** | **MASDFNLPPVVAKEI** | **0.33** |
|  |  |  | **KEGHIAKNCRAPRKK** | **1.03** | **GKIILVAVHVASGYI** | **0.16** |
|  |  |  | **RKKGCWKCGKEGHQM** | **0.27** | **HVASGYIEAEVIPAE** | **0.34** |
|  |  |  | **GKEGHQMKDCTERQA** | **0.23** | **AEVIPAETGQETAYF** | **0.15** |
|  |  |  | **DCTERQANFLGKIWP** | **0.80** | **GQETAYFLLKLAGRW** | **0.18** |
|  |  |  | **IWPSHKGRPGNFLQS** | **0.84** | **HTDNGSNFTSTTVKA** | **1.17** |
| **CP-5** | **RERMRRAEPAADGVG** | **0.73** | **QAISPRTLNAWVKVV** | **0.36** | **HRTKIEELRQHLLRW** | **0.56** |
|  | **QVPLRPMTYKAAVDL** | **0.47** | **SALSEGATPQDLNTM** | **2.30** |  |  |
|  | **FPDWQNYTPGPGIRY** | **0.18** | **GHKARVLAEAMSQVT** | **0.17** |  |  |
|  | **IRYPLTFGWCFKLVP** | **0.48** |  |  |  |  |
| **CP-6** | **GAITSSNTAANNADC** | **0.4** | **EKIRLRPGGKKKYKL** | **0.52** | **RWKPKMIGGIGGFIK** | **0.56** |
|  | **ADCAWLEAQEEEEVG** | **0.32** | **YKLKHIVWASRELER** | **0.23** | **FIKVRQYDQILIEIC** | **0.78** |
|  | **QEEEEVGFPVRPQVP** | **0.28** | **GLNKIVRMYSPTSIL** | **3.64** | **IGRNLLTQIGCTLNF** | **0.72** |
|  | **PVRPQVPLRPMTYKA** | **1.13** | **IRQGPKEPFRDYVDR** | **0.12** | **IRYQYNVLPQGWKGS** | **0.46** |
|  |  |  | **FRDYVDRFYKTLRAE** | **0.70** | **KGSPAIFQSSMTKIL** | **0.29** |
|  |  |  | **ASQEVKNWMTETLLV** | **0.25** | **PFRKQNPDIVIYQYM** | **0.49** |
|  |  |  |  |  | **IVIYQYMDDLYVGSD** | **0.4** |
|  |  |  |  |  | **KRKGGIGGYSAGERI** | **0.17** |
|  |  |  |  |  | **ERIVDIIATDIQTKE** | **0.24** |
|  |  |  |  |  | **TKELQKQITKIQNFR** | **0.81** |
|  |  |  |  |  | **NFRVYYRDSRDPLWK** | **0.35** |
| **CP-7** | **HFLKEKGGLEGLIYS** | **1.28** | **LGPAATLEEMMTACQ** | **0.17** | **SINNETPGIRYQYNV** | **0.86** |
|  | **VYHTQGYFPDWQNY** | **0.33** | **EAMSQVTNSATIMMQ** | **0.54** | **IRYQYNVLPQGWKGS** | **0.16** |
|  |  |  |  |  | **IVIYQYMDDLYVGSD** | **0.23** |
|  |  |  |  |  | **DLYVGSDLEIGQHRT** | **0.18** |
|  |  |  |  |  | **WEAWWTEYWQATWIP** | **0.26** |
|  |  |  |  |  | **WEFVNTPPLVKLWYQ** | **0.15** |
|  |  |  |  |  | **IVGAETFYVDGAANR** | **1.18** |
|  |  |  |  |  | **NQKTELQAIHLALQD** | **0.05** |
|  |  |  |  |  | **GLEVNIVTDSQYALG** | **0.17** |
|  |  |  |  |  | **IQAQPDKSESELVSQ** | **0.19** |
|  |  |  |  |  | **KVYLAWVPAHKGIGG** | **0.25** |
